# Efficacy of *Stb* resistance genes and pathotype diversity in *Zymoseptoria tritici* from Ethiopia

**DOI:** 10.1038/s41598-025-13035-x

**Published:** 2025-07-31

**Authors:** Ayantu Tucho, Tilahun Mekonnen, Kassahun Tesfaye, Diriba Muleta, Tesfaye Alemu, Farideh Ghadamgahi, Aakash Chawade, Ramesh Raju Vetukuri

**Affiliations:** 1 Department of Plant Science, Salale University, P. O. Box 245, Fitche, Ethiopia; 2 Institute of Biotechnology, Addis Ababa University, Box 1176, Addis Ababa, Ethiopia; 3 Bio and Emerging Technology Institute (BETin), P. O. Box 5954, Addis Ababa, Ethiopia; 4 Department of Microbial, Cellular and Molecular Biology, Addis Ababa University, Addis Ababa, Ethiopia; 5 Department of Plant Breeding, Swedish University of Agricultural Sciences, P. O. Box 102, Alnarp, 230 53 Sweden

**Keywords:** Aggressiveness, Necrotic leaf area, Pycnidia coverage, *Stb* gene, *Zymoseptoria tritici*, Plant breeding, Plant immunity, Plant stress responses, Fungi, Pathogens

## Abstract

*Zymoseptoria tritici*, the causal agent of Septoria tritici blotch (STB) in bread wheat (*Triticum aestivum*), leads to significant global yield losses. Resistance breeding is vital for managing STB, but there is limited information on *Z. tritici* infection behaviour in Ethiopia. This study examined the virulence variability of *Z. tritici* isolates from Ethiopia’s Central Highlands and evaluated the effectiveness of known wheat STB-resistance genes. Eight wheat lines were tested against six *Z. tritici* isolates, showing significant differences (*p* < 0.0001) in necrotic leaf area (%NLA) and pycnidia coverage (%PC) among the tested *Z. tritici* isolates, wheat lines and their interactions. Wheat genotype TE9111 exhibited specific resistance to 50% of the isolates, while Taichung 29 showed no resistance. Isolate ZSE158 was the most aggressive, causing 61.4% PC and 54% NLA. The Ethiopian isolates displayed broad virulence against resistance genes, including Stb2 – Stb7. TE9111, carrying Stb11, showed resistance to 50% of isolates, making it a valuable source for resistance breeding against STB. This study identified highly virulent pathogen isolates useful for wheat germplasm screening for STB resistance and also key resistance source materials for use in wheat resistance breeding in Ethiopia.

**Supplementary Information:**

The online version contains supplementary material available at 10.1038/s41598-025-13035-x.

## Introduction

Wheat (*Triticum aestivum*) is a crucial staple food crop in Ethiopia, contributing significantly to food and nutritional security. The country’s diverse agro-ecologies allow for wheat cultivation across various regions, making it an essential crop for both smallholder farmers and commercial production systems. In Ethiopia, wheat ranks fourth after teff (*Eragrostis tef*), maize (*Zea mays*) and sorghum (*Sorghum bicolor*) in area coverage, and third after maize and teff in total production^1^. It is cultivated by more than 5 million householders on about 2.3 million ha for various uses, such as food, feed and income generation. Both durum and bread wheat are cultivated in Ethiopia, the latter accounting for nearly 80% of the country’s total wheat production^2,3^. During the last 15 years, wheat harvest area, production and productivity in Ethiopia have increased from 1.4 M ha, 2.31 million metric tons and 1.62 t/ ha in 2003, to 2.3 M ha, 7 million metric tons and 3.04 ton/ha in 2022, respectively^4^. However, this national average wheat productivity (3.04 t/ha) is far lower than the global average of 3.69 t/ha^5^, resulting in a production limit to meeting the growing demand for food by an ever-increasing population^6^. Wheat production in Ethiopia can be curtailed by several factors, such as limited access to advanced agricultural production technologies and low agricultural inputs (improved varieties and fertilizer), biotic stresses such as disease, pests and weeds, and abiotic stresses including drought, soil acidity and salinity^7^.

Among the biotic factors, diseases caused by fungal pathogens represent the major constraints affecting wheat production and productivity globally. In Ethiopia, wheat cultivation is persistently affected by over 30 fungal diseases^8^, among which stripe rust (yellow rust) caused by *Puccinia striiformis* f. sp. *tritici*, stem rust caused by *Puccinia graminis* f. sp. *tritici*, and Septoria tritici blotch (STB) caused by *Zymoseptoria tritici* are the major infections^8–10^. Rusts can result in grain yield losses of 60–100%^11^. Up to 82% losses in wheat yield have been reported to be the result of STB, and currently the prevalence of STB has increased considerably in the major wheat-growing areas of Ethiopia^12,13^. STB affects grain yield by causing reduced tillering, poor seed set, poor grain fill or shriveled kernels, and the death of leaves, spikes or the entire plant^14,15^ .

The development and use of resistant varieties is the most economical, durable and environmentally safe approach to controlling crop diseases^16^. The gene pool utilized in wheat breeding efforts has expanded as a result of extensive research into additional sources of STB resistance^17^. Similar to many other plant diseases, wheat has two types of STB resistance: qualitative and quantitative. Quantitative resistance is regulated by polygenic characteristics and provides insufficient resistance to *Z. tritici*. In contrast, qualitative resistance confers complete or near complete resistance to particular isolates and follows a gene-for-gene model. So far, 22 resistance genes in *Z. tritici* have been reported and mapped on the wheat genome^17–19^. However, the expression patterns and effects of these genes on STB resistance vary between the seedling and adult plant stages^20^. For example, *Stb16* is expressed and effective at both seedling and adult stages, while *Stb17* is only expressed during the adult stage^21^. *Stb18* is an isolate-specific resistance gene, displaying varying resistance to *Z. tritici* depending on the isolate, in both seedling and adult stages^21^. *Stb6* is the only qualitative gene for STB resistance^22^, and its corresponding avirulence gene, *AvrStb6*, in *Z. tritici*^23^ has been shown to follow a gene-for-gene relationship. However, because of its reproductive biology (sexual life cycle), *Z. tritici* changes its genome rapidly, favoring adaptions to host resistance genes (*R* genes)^24^. In line with this^25^, suggest that the narrow genetic basis of modern wheat cultivars and the rapidly changing fungal genomes have together resulted in the frequent breakdown of host resistance.

Disease can happen when the balance between a host and a pathogen is altered. Thus, host defense can never be considered independent of a pathogen’s virulence factor. In other words, effective resistance breeding relies on a clear understanding of the disease-causing Pathotype and its genetic structure. The term ‘Pathotype’ describes populations of a fungus species that are recognized as having identical morphology but distinct infection behaviors, which may be distinguished by the way the populations respond to a series of test host cultivars called ‘differentials’ ^26,27^ used wheat differential lines with known *Stb* genes to assess virulence variability in *Mycosphaerella graminicola* and *Z. tritici* populations. Screening of seedlings based on the gene-for-gene concept provides the opportunity to determine the effectiveness of resistance against a broad range of isolates^21^.

Despite the greater incidence and severity of STB disease on wheat, there is limited information on the infection behavior of *Z. tritici* populations in Ethiopia. Therefore, the purpose of this study was to evaluate the effectiveness of Stb resistance genes and assess the pathotype diversity of Zymoseptoria tritici isolates from Ethiopia through artificial inoculation under greenhouse conditions.

## Results

### Integrative analysis of variance

Variance analysis is used to assess the significance of isolate-by-genotype interactions within the plant–pathogen relationship and identify differential outcomes. Six *Z. tritici* isolates were chosen from a pool of 200, by comparing the isolates identified in a phylogenetic tree following internal transcribed spacer (ITS) region sequencing in accordance with the location of their collection. The infection behavior of the six *Z. tritici* isolates was assessed on eight wheat differential genotypes with known *Stb* genes (Table 1). Plants with a score of 0–2 were categorized as resistant, while those with a score of 3–5 were considered susceptible. The data on disease severity revealed highly significant differences (*p* < 0.0001) (Table 1) in the percentage of necrotic leaf area (%NLA) and pycnidia coverage (%PC) between the isolates, wheat differential lines, and their two-way interactions (Table 2). The symptoms of each genotype was compared with Taichung 29 used as susceptible control. The variation based on wheat genotypes was the largest. Based on an ANOVA, the isolates’ main effect was also significant (Table 1), suggesting that Ethiopian *Z. tritici* isolates have significant variability in their virulence, providing the second highest source of variation. Similarly, %NLA and %PC values were significantly different (*p* < 0.0001) among the wheat differential lines, indicating that the genotypes differed greatly in their responses to the *Z. tritici* isolates. Furthermore, the extremely significant differences in the isolate-by-genotype interactions indicated the existence of specificity among the wheat differential lines to the pathogen inoculum, and also the presence of considerable differences among the pathogen inoculum. This suggested a distinct interaction between the genotypes of the isolates and hosts.

**Table 1:** A summary of an ANOVA showing the percentages of necrotic leaf area (NLA) and pycnidia coverage (PC) caused by six Ethiopian *Zymoseptoria tritici* isolates on eight wheat differential lines.

Source	DF	Mean square	
		%NLA	%PC
Wheat genotype	7	7907.33^***^	7153.24^***^
Fungal isolates	5	640.68^***^	763.711^***^
Genotype × isolate	35	317.79^***^	287.54^**^
*R* ^2^		0.87	0.87
CV (%)		18.20	15.20
Root MSE		8.66	8.29

***Very highly significant (*p* < 0.0001); DF = degrees of freedom, CV = Coefficient of Variance, MSE = Mean Square Error.

**Table 2:** Percentage pycnidia coverage (PC) and necrosis leaf area (NLA) on eight wheat differential lines genotype due to *Zymoseptoria tritici infection*.

Wheat genotype	Z. tritici isolate													
	ZSET158		ZSET168		ZSET121		ZSET206		ZSET033		ZSET218		Mean ^a^	
	PC	NLA	PC	NLA	PC	NLA	PC	NLA	PC	NLA	PC	NLA	PC	NLA
Estanzuela Federal (*Stb7*)	47	41	48	38	58	45	50	39	47	37	42	32	48	39
Israel 493	72	64	60	54	40	38	51	43	63	59	63	60	58	53
Shafir (*Stb6*)	71	60	64	49	66	59	65	57	58	49	62	54	64	55
TE9111	14^*^	11^**^	10^**^	6^*^	31	25	30	19	26	18	13^*^	8^*^	21	15
Tadinia	66	61	56	48	58	51	63	60	55	50	39	13^*^	56	49
Taichung 29	85	77	76	71	84	84	82	83	85	80	81	78	82	79
CS/synthetic (6x)	64	52	67	62	45	37	11^**^	37	45	23	27	15^*^	46	38
Veranopolis	67	57	57	46	53	49	60	60	60	50	42	40	57	50
Mean^a^	61	53	55	47	54	48	56	50	53	46	46	39		
PC–LSD_5%_^b^ = 11.584 NLA–LSD_5%_^b^ = 12.097 LSD_1%_^b^ = 15.299 LSD_1%_^b^ = 15.976														

^a^ Mean disease scores were calculated by omitting data for specific interactions.

^b^ Least significant difference (LSD) between means of disease scores.

* Highly resistant = not significantly different from zero (according to LSD5%).

**Resistant = not significantly different from zero (according to LSD1%).

PC = Pycnidia coverage (%).

NLA = Necrotic leaf area (%).

### Pathogenicity, aggressiveness and virulence of *Z. tritici* isolates

**Fig. 1:**
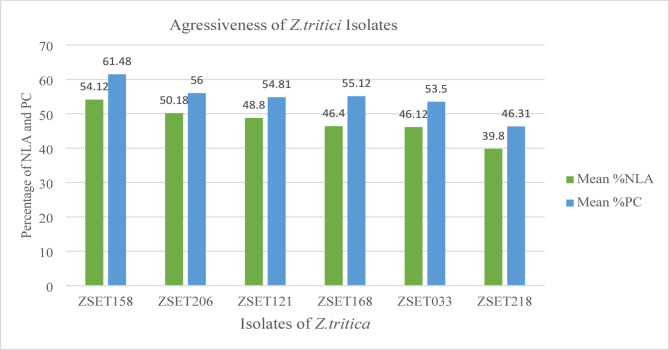
The mean percentage necrotic leaf area (NLA) and pycnidia coverage (PC) caused by six *Zymoseptoria tritici* isolates tested on eight wheat genotypes grouped by Tukey grouping for mean of isolate at Alpha = 0.05.

**Fig. 2:**
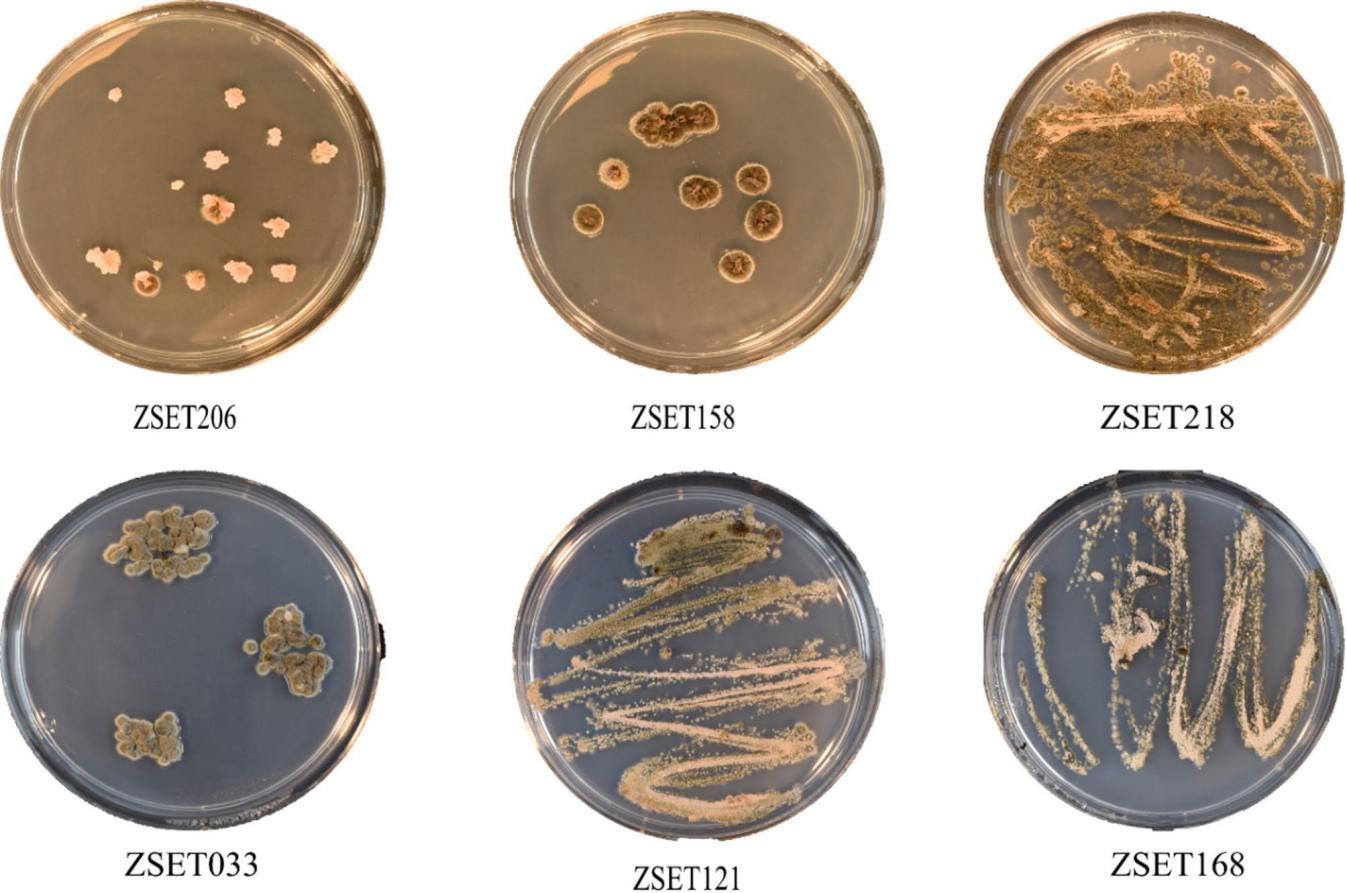
Colonies of six different *Zymoseptoria tritici* isolates cultured on potato dextrose agar (PDA) and inoculated on eight wheat differential lines.

**Fig. 3:**
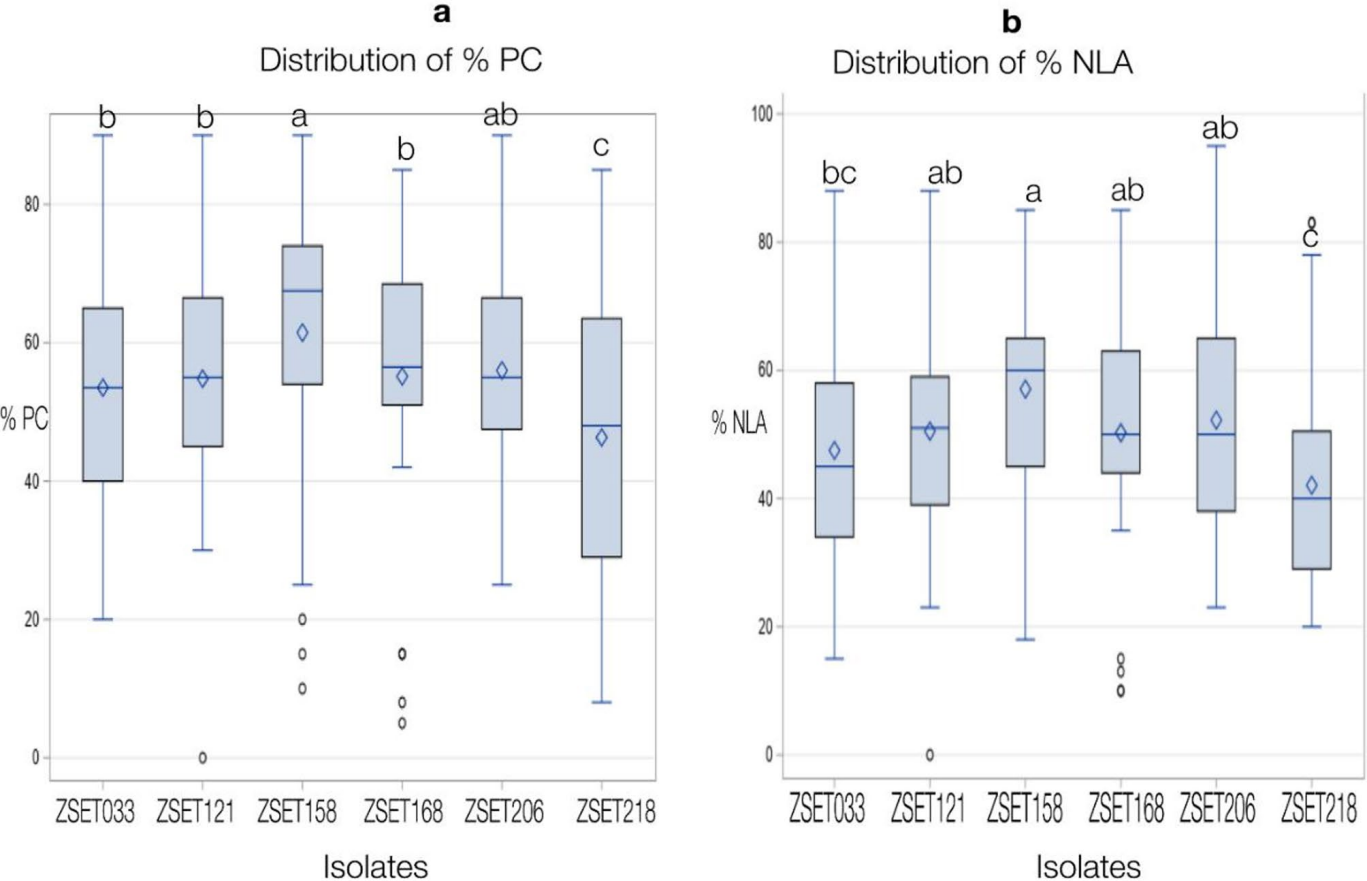
Boxplots of necrotic leaf area (NLA, **a**) and pycnidia coverage (PC, **b**) caused by six *Zymoseptoria tritici* isolates on eight wheat Septoria differential lines.

Aggressiveness is a pathogen’s relative capacity to infect a host, and is a quantitative aspect of pathogenicity, whereas virulence is a distinguishable isolate-specific relationship^28^. Differential interactions always relate to virulence (which is linked to the presence or absence of *R* genes), whereas ‘aggressiveness’ describes a quantitative component of pathogenicity that is, by definition, non-specific relative to host genotypes. The classic susceptible wheat cultivar, Taichung 29, demonstrated high virulence for all isolates. Examination of the isolates’ aggressiveness showed that the wheat genotypes varied significantly in their response to them (manifest as mean disease severity). The *Z. tritici* isolates tested displayed broad-spectrum virulence across the wheat genotypes. Isolate ZSE158 was the most aggressive, with the highest mean PC of 61.4% and NLA of 54% (Fig. 1.). With a mean PC of 56% and NLA of 50%, ZSET206 was the second most aggressive. In contrast, isolate ZSET218 was the least aggressive, with a mean PC and NLA of 46% and 39.8%, respectively (Figs. 1, 2 and 3).

### Resistance spectra for wheat septoria differential lines against Ethiopian *Z. tritici* isolates

**Fig. 4:**
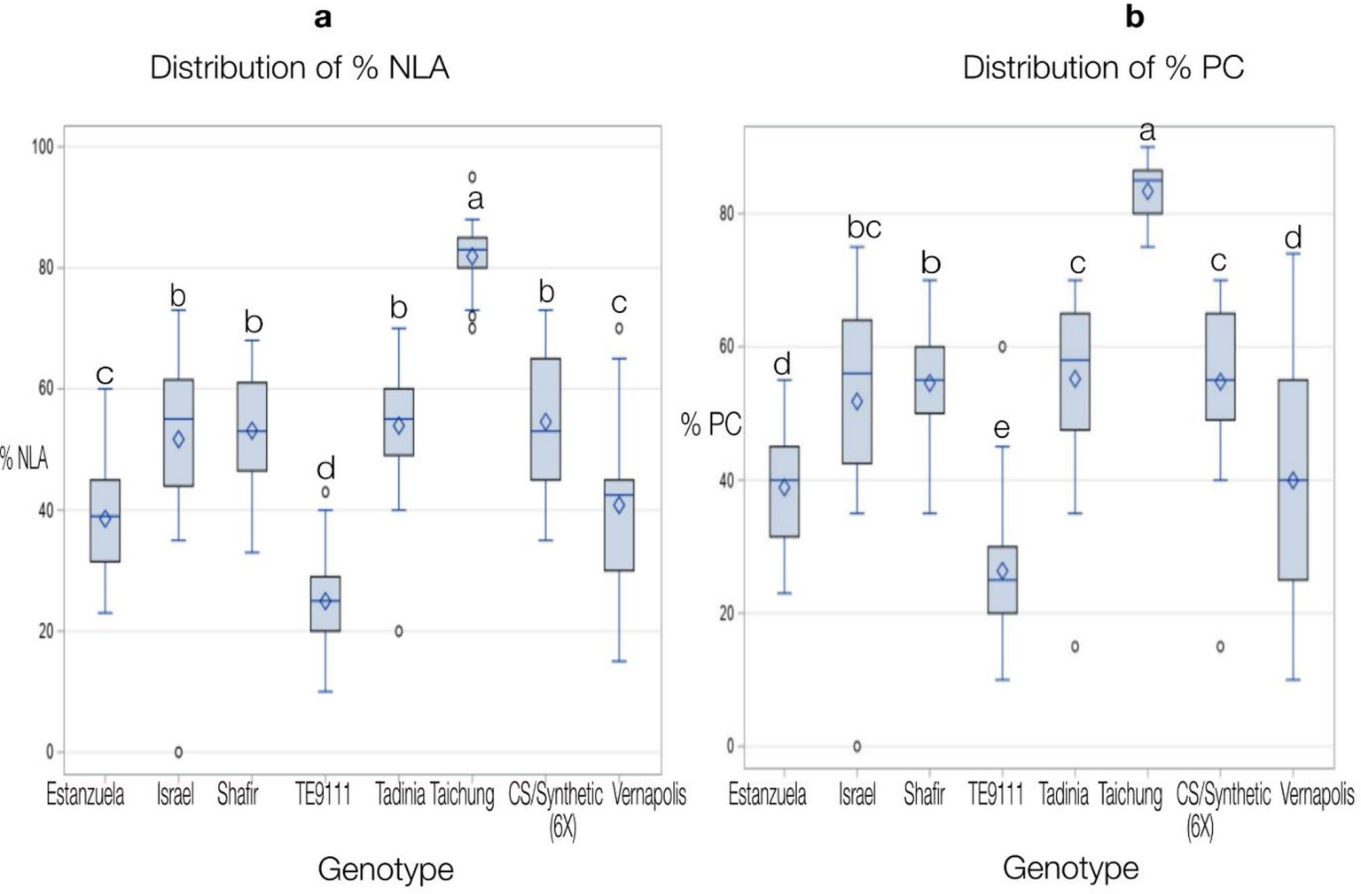
The distribution of necrotic leaf area (NLA, **a**) and pycnidia coverage (PC, **b**) caused by six *Zymoseptoria tritici* isolates on eight wheat Septoria differential lines.

The ANOVA results showed that the wheat differential lines, isolates, and their two-way interactions, varied significantly in terms of disease severity (%PC and %NLA) (Table 2). Among 48 isolate-by-wheat genotype interactions, four (8.3%) and five (10.4%) showed mean PC and NLA values lower than the LSD values at *p* < 0.01 and *p* < 0.05 levels, respectively, and thus they could be considered resistant to one or more of the tested *Z. tritici* isolates (Table 3). Among the wheat genotypes used in this study, TE9111, which carries *Stb11*, was resistant to three (50%) *Z. tritici* isolates (Table 3; Fig. 4). Likewise, the genotypes CS Synthetic and Tadinia showed were resistant to two and one isolates, respectively (Table 3).

**Table 3:** The reaction of known *Stb* differentials to six Ethiopian *Zymoseptoria tritici* isolates.

Wheat differential line	Stb gene	Z. tritici isolate					
		ZSET158	ZSET168	ZSET206	ZSET121	ZSET033	ZSET218
Veranopolis	*Stb2* + *Stb6*	S	S	S	S	S	S
Israel 493	*Stb3* + *Stb6*	S	S	S	MS	S	S
Tadinia	*Stb4* + *Stb6*	S	S	S	S	S	MS
CS Synthetic (6x)	*Stb5* + *Stb6*	S	S	R	S	S	MR
Shafir (*Stb6*)	*Stb6*	S	S	S	S	S	S
Estanzuela Federal (*Stb7*)	*Stb7*	S	S	S	S	S	S
TE9111	*Stb11*	R	HR	S	MS	MR	HR
Taichung 29	Susceptible control	S	S	S	S	S	S

*Stb* = Septoria-resistant genes in differential lines; HR = highly resistant; R = resistant; MR = moderately resistant; MS = moderately susceptible; S = susceptible; VS = very susceptible.

**Fig. 5:**
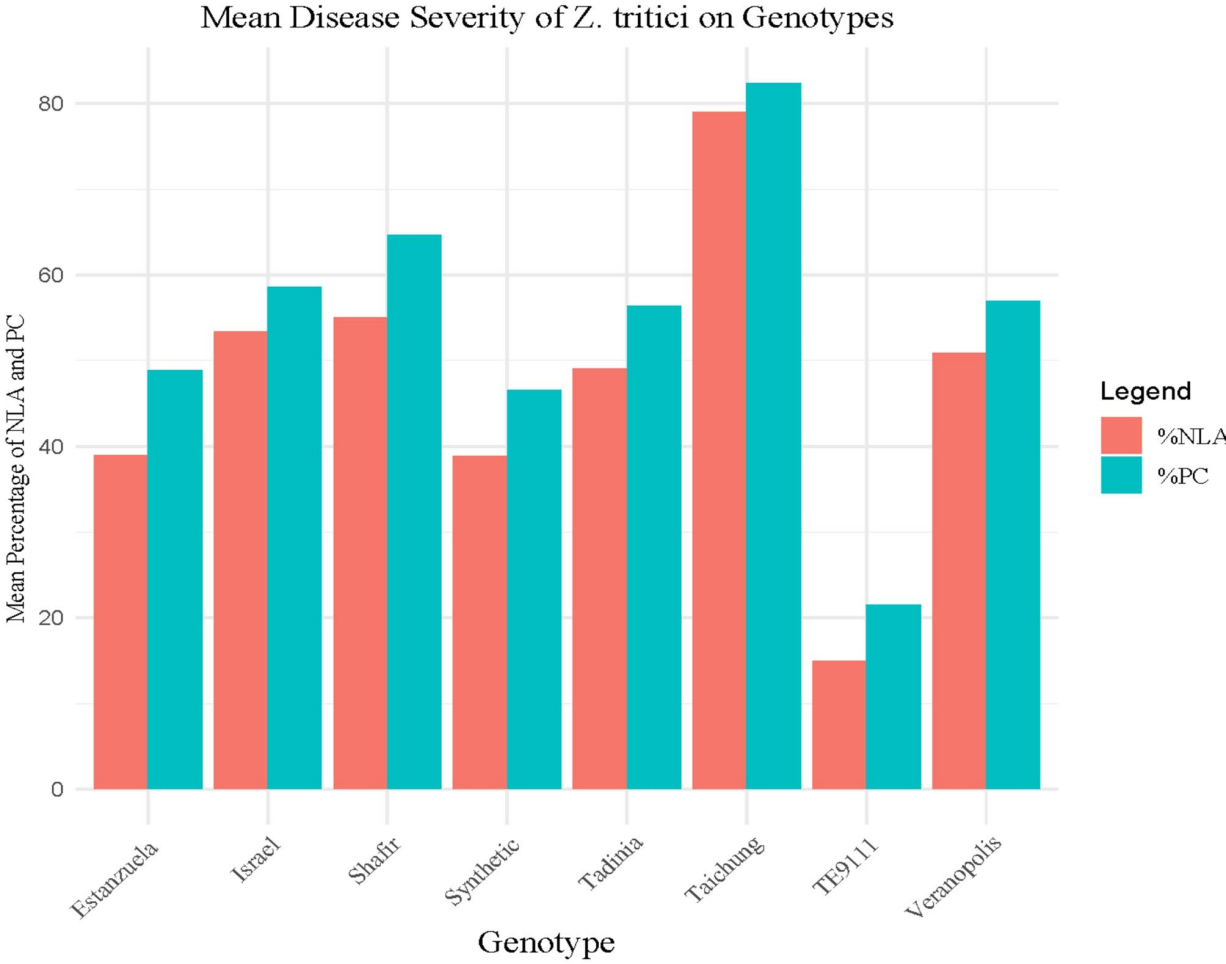
Mean of eight wheat differential lines based on percentage mean disease severity (necrotic leaf area, %NLA, pycnidia coverage, %PC) caused by six *Zymoseptoria tritici* isolates by Tukey mean grouping at Alpha = 0.05 significance level.

Five of the tested differential lines, Estanzuela Federal, Israel 493, Shafir (*Stb6*), Taichung 29 and Veranopolis, showed 100% susceptibility to the tested *Z. tritici* isolates (Table 3). None of the tested wheat genotypes showed resistance to all the *Z. tritici* isolates used. Only TE9111 and CS Synthetic (6x) showed significant specific resistance to one or more of the tested *Z. tritici* isolates, suggesting that they possessed one or more *Stb* genes effective against a limited number of *Z. tritici* isolates (Table 3). Among the tested wheat differential lines, TE9111 and Taichung 29 were found to be the most resistant and susceptible, respectively (Table 3; Fig. 5 and Table 4).

**Table 4:** Mean percentage of necrotic leaf area (NLA) and pycnidia coverage (PC) measured on eight wheat differential lines infected with six *Zymoseptoria tritici* isolates of Ethiopia.

No	NLA Tukey grouping for genotype × isolate LSM (alpha = 0.05)			PC Tukey grouping for genotype × isolate LSM (alpha = 0.05)		
	Genotype	Isolate	LSM	Genotype	Isolate	LSM
1	Taichung	ZSET121	84^a***^	Taichung	ZSET033	85^a***^
2	Taichung	ZSET206	83^ab^	Taichung	ZSET158	85^a***^
3	Taichung	ZSET033	80^a − c^	Taichung	ZSET121	84^ab^
4	Taichung	ZSET218	78^a − d^	Taichung	ZSET206	82^a − c^
5	Taichung	ZSET158	77^a − d^	Taichung	ZSET218	81^a − d^
6	Taichung	ZSET168	71^a − e^	Taichung	ZSET168	76^a − e^
7	Israel	ZSET158	64^a − f^	Israel	ZSET158	72^a − f^
8	CS/Synthetic (6X)	ZSET168	62^a − g^	Shafir	ZSET158	71^a − g^
9	Tadinia	ZSET158	61^a − h^	CS/Synthetic(6X)	ZSET168	67^b − h^
10	Veranopolis	ZSET206	61^a − h^	Veranopolis	ZSET158	67^b − h^
11	Israel	ZSET218	61^a − h^	Tadinia	ZSET158	66^b − h^
12	Shafir	ZSET158	61a-h	Shafir	ZSET121	66^b − i^
13	Tadinia	ZSET206	60^a − h^	Shafir	ZSET206	65^b − j^
14	Shafir	ZSET121	59^a − h^	Shafir	ZSET168	64^b − k^
15	Israel	ZSET033	59^b − h^	CS/Synthetic (6X)	ZSET158	64^b − k^
16	Shafir	ZSET206	58^c − h^	Israel	ZSET033	63^b − k^
17	Veranopolis	ZSET158	57^d − h^	Tadinia	ZSET206	63^b − k^
18	Israel	ZSET168	54 ^d−i^	Israel	ZSET218	63^b − k^
19	Shafir	ZSET218	54^d − i^	Shafir	ZSET218	62^b − l^
20	CS/Synthetic (6X)	ZSET158	52^e − i^	Veranopolis	ZSET206	60^b − l^
21	Tadinia	ZSET121	51^e − i^	Israel	ZSET168	60^b − l^
22	Veranopolis	ZSET033	51^e − i^	Veranopolis	ZSET033	60^d − l^
23	Tadinia	ZSET033	50^e − j^	Shafir	ZSET033	58^d − l^
24	Shafir	ZSET033	50^e − j^	Estanzuela	ZSET121	58^d − l^
25	Veranopolis	ZSET121	49^e − j^	Tadinia	ZSET121	58^d − l^
26	Shafir	ZSET168	49^e − j^	Veranopolis	ZSET168	57^d − l^
27	Tadinia	ZSET168	48^e − k^	Tadinia	ZSET168	56^e − l^
28	Veranopolis	ZSET168	46^f − l^	Tadinia	ZSET033	55^e − m^
29	Estanzuela	ZSET121	45^f − l^	Veranopolis	ZSET121	53^e − m^
30	Israel	ZSET206	43^f − l^	Israel	ZSET206	51 ^i−n^
31	Estanzuela	ZSET158	41^g − l^	Estanzuela	ZSET206	50^i − o^
32	Veranopolis	ZSET218	40^g − l^	Estanzuela	ZSET168	48^i − p^
33	Estanzuela	ZSET206	39^g − l^	Estanzuela	ZSET158	47^i − p^
34	Israel	ZSET121	38^g − n^	Estanzuela	ZSET033	47^i − p^
35	Estanzuela	ZSET168	38^g − n^	CS/Synthetic (6X)	ZSET121	45^i − p^
36	Estanzuela	ZSET033	37^h − n^	CS/Synthetic (6X)	ZSET206	45^i − p^
37	CS/Synthetic (6X)	ZSET121	37^h − n^	Veranopolis	ZSET218	42^i − p^
38	CS/Synthetic (6X)	ZSET206	37^h − n^	Estanzuela	ZSET218	42^j − p^
39	Estanzuela	ZSET218	33^i − n^	Israel	ZSET121	40^k − q^
40	TE9111	ZSET121	25^j − p^	Tadinia	ZSET218	39^l − q^
41	Tadinia	ZSET218	23^l − p^	TE9111	ZSET121	31^m − q^
42	CS/Synthetic (6X)	ZSET033	23^l − p^	CS/Synthetic (6X)	ZSET033	31^n − q^
43	CS/Synthetic (6X)	ZSET218	22^l − p^	TE9111	ZSET206	30^n − q^
44	TE9111	ZSET206	20^m − p^	CS/Synthetic (6X)	ZSET218	27^o − r^
45	TE9111	ZSET033	19^n − p^	TE9111	ZSET033	26^q − r^
46	TE9111	ZSET158	11^op^	TE9111	ZSET158	17^qr^
47	TE9111	ZSET218	8^op^	TE9111	ZSET218	13^r***^
48	TE9111	ZSET168	6^p***^	TE9111	ZSET168	11^r***^

Least square means (LSM) with the same letter are not significantly different. *** = very highly significant.

### Cluster analysis of wheat genotypes

**Fig. 6:**
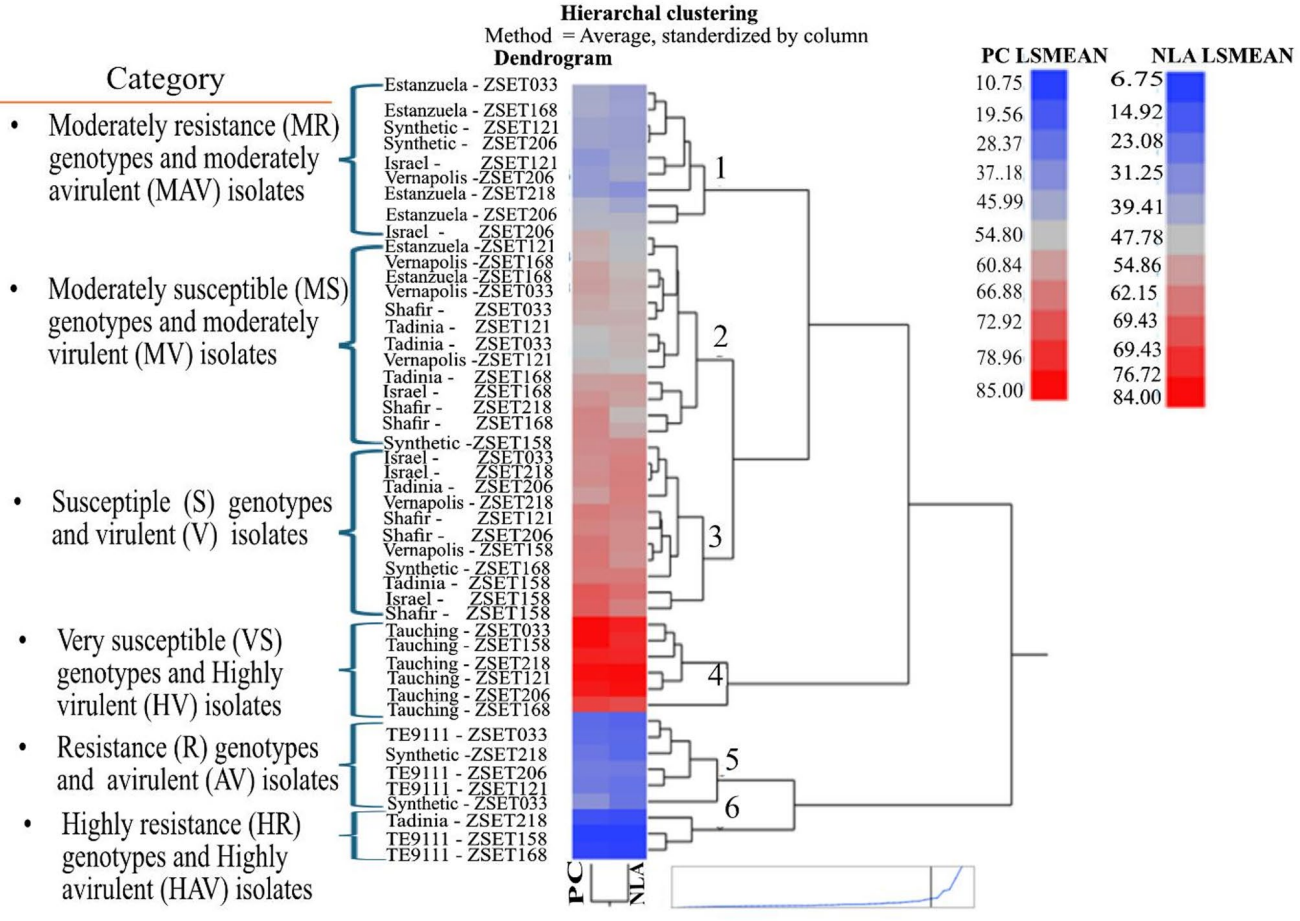
Hierarchal clustering of 48 *Zymoseptoria tritici* isolate – wheat genotype interactions based on mean disease severity (necrotic leaf area, NLA, and pycnidia coverage, PC).

Hierarchical cluster analysis based on mean disease severity (NLA and PC) grouped the eight differential lines (wheat genotypes) into six clusters (Fig. 6). Cluster I contained nine (18.75%) of the pairwise isolate-by-genotype interactions; the genotypes were categorized as moderately resistant (MR) and the isolates as moderately avirulent (MAv). Cluster II comprised 13 (27.08%) of the interactions, which were considered to have moderately susceptible (MS) genotypes and moderately virulent (MV) isolates. Cluster III contained 11 (22.9%) interactions, which were categorized as susceptible (S) genotypes and virulent (V) isolates. Cluster IV contains six (12.5%) genotype-by-isolate combinations that comprise very susceptible (VS) genotypes and highly virulent (HV) isolates. Cluster V contained six (12.5%) combinations categorized as resistance (R) genotypes and avirulent (Av) isolates. Cluster VI consisted of three (6.25%) combinations that were categorized as highly resistance (HR) genotypes and highly avirulent (HAv) isolates.

### Efficacy of *Stb*-resistant genes against Ethiopian *Z. tritici* isolates

**Fig. 7:**
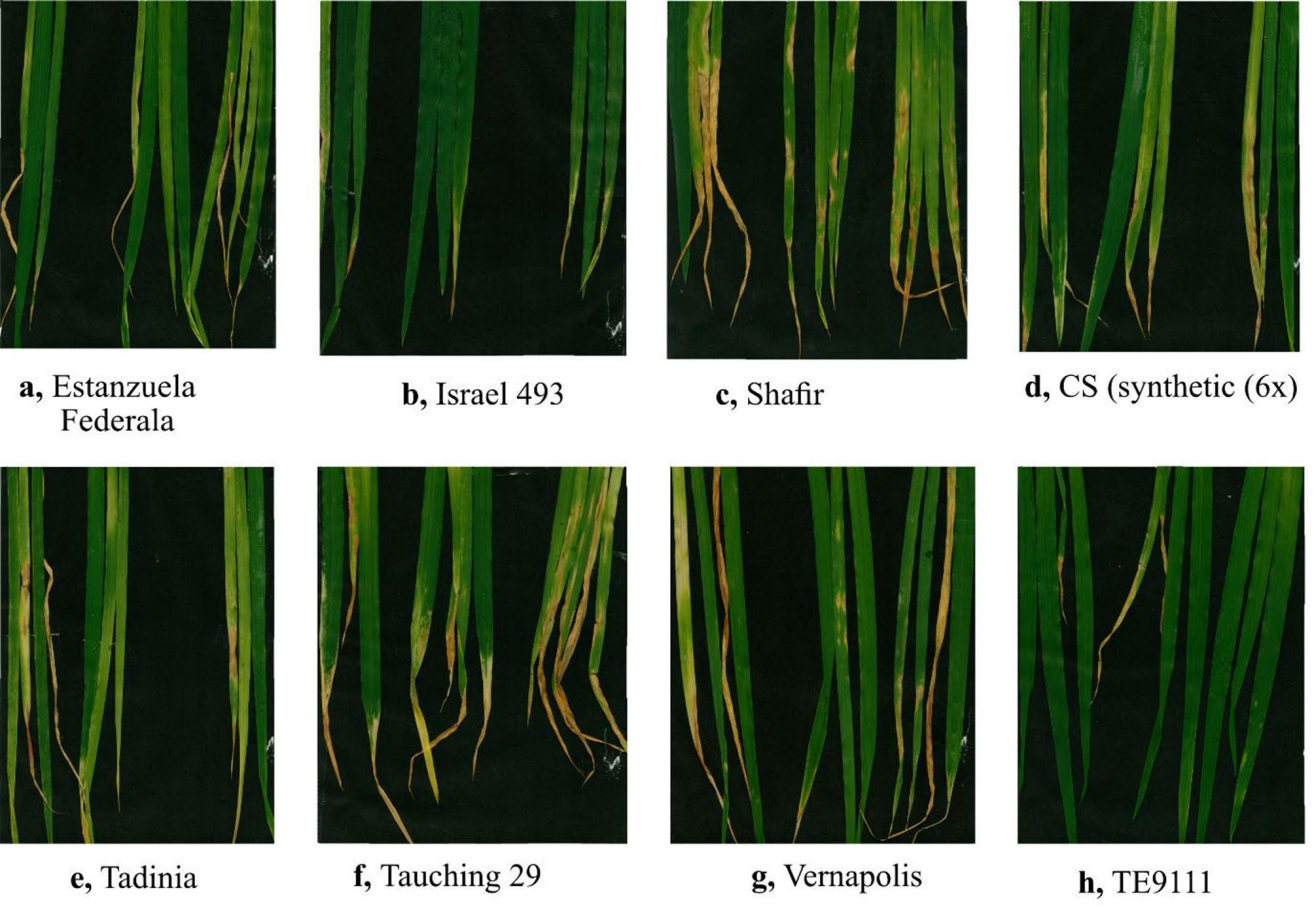
Septoria tritici blotch developed by the *Z. tritici* isolate ZSET158 on eight Septoria wheat differential lines: (**a**) Estanzuela; (**b**) Israel 493; (**c**) Shafir; (**d**) CS/Synthetic (6X); (**e**) Tadinia; (**f**) Taichung 29 (susceptible control); (**g**) Veranopolis; and (**h**) TE9111.

Table 3 presents a summary of the virulence level of the six *Z. tritici* isolates on the eight differential lines with known major *Stb* genes. All six isolates showed variations in their virulence against the *Stb* genes. Accordingly, TE9111, which possessed *Stb11*, was highly resistant (HR) to the isolates ZSET168 and ZSET218, moderately resistant (MR) to ZSET033, moderately susceptible (MS) to ZSET121, and susceptible (S) to ZSET206. Genotype CS Synthetic (6x), which possessed *Stb5* + *Stb6*, showed resistance (R) to the isolate ZSET206, and moderate resistance (MR) to ZSET218, but was susceptible (S) to all other *Z. tritici* isolates. The differential lines Israel 493 and Tadinia were found to be moderately susceptible to ZSET121 and ZSET218, respectively. The other wheat differential lines, such as Veranopolis (*Stb2* + *Stb6*), Shafir (*Stb6*), Estanzuela Federal (*Stb7*) and the susceptible control Taichung 29 (*Stb2* + *Stb6*, *Stb6*, *Stb7*), were found to be totally susceptible to the Ethiopian *Z. tritici* isolates. Among the *Stb* genes, Stb11 in TE9111 was the most effective, conferring resistance to four *Z. tritici* isolates (Table 3). Differential lines with known *Stb* genes that scored mean disease severity values higher than the critical LSD values were considered as susceptible and designated S, while those with mean disease severity values (NLA and PC) lower than the LSD values at α = 1% were considered resistant and denoted as R (Table 4). Figure 7 illustrates the resistance response of the eight wheat Septoria differential lines with known *Stb* genes against the *Z. tritici* isolate ZSET158. The resistant Israel 493 and TE9111 lines developed no/minimum symptoms of STB infection compared with susceptible lines. This was due to that TE9111 differential was the first resistant variety and Israel was the second resistant variety according to the result of the investigation.

## Discussions

This study evaluated the efficacy of known Stb resistance genes and assessed the Pathotype diversity of *Zymoseptoria tritici* isolates collected from Ethiopia. The findings highlight significant variability in both the effectiveness of Stb genes and the virulent patterns of *Z. tritici* populations, emphasizing the dynamic nature of host-pathogen interactions in wheat production.

The virulence patterns of six *Z. tritici* isolates (Fig. 7) obtained from Ethiopia’s major wheat-growing areas were examined on eight differential lines of bread wheat (Table 7) that possessed different *Stb* genes. Using %PC and %NLA as disease parameters^16,29^, the results provide relevant information for wheat breeders regarding the virulence spectrum and aggressiveness of Ethiopian populations of *Z. tritici* isolates, as well as the response of previously reported *Stb* genes. In a study conducted by^30^ in Ethiopia, several *Z. tritici* isolates were found to be virulent on the differential lines such as Veranopolis, Tadinia, Km 7 and Kavkaz-K4500, out of the seven tested. Similarly^31^ reported that six isolates out of eight tested isolates were virulent on the genotypes tested. This finding is similar to the current results. Collectively, the isolates from both the present study and the earlier investigation demonstrate that Ethiopian *Z. tritici* populations are capable of overcoming resistance in differential lines tested which was previously considered one of the most resistant differential lines.

The combined analysis of variance for both parameters (%PC and %NLA) revealed extremely significant differences (*p* < 0.0001) in the interaction effects, indicating that the isolates varied considerably in their virulence patterns across the wheat genotypes (Table 1). The particular interactions between isolates and genotypes^32^ verify the presence of genotype specificity and virulence diversity in Ethiopian *Z. tritici* isolates (Table 2). Among the 48 isolate-by-genotype interactions, four (8.3%) and five (10.4%) had mean %PC and %NLA values lower than the LSD values at *p* < 0.01 and *p* < 0.05 significance levels, respectively (Table 4), indicating that these interactions could be focus points for sources of resistance.

These results are consistent with^31^, who reported highly significant differences between wheat genotypes, *Z. tritici* and their interactions, confirming that Ethiopian *Z. tritici* populations display broad spectrum virulence. The findings of the current study confirm that the isolates possess broad-spectrum virulence, making them valuable tools for use in wheat resistance breeding programs, particularly for screening germplasm against diverse Pathotype which is similar result with previous study by^30^ in the same country. Likewise^33^, identified highly significant levels of virulence diversity among Iranian Z. *tritici* isolates, ranging from 40 to 90%, and^34^ have demonstrated the existence of substantial virulence differences among *Z. tritici* isolates in Iran.

Among the tested wheat genotypes, TE9111 exhibited specific resistance to three isolates (50%) (Fig. 4.), indicating that it is a promising source of resistance against STB for use in future wheat breeding programs^35^^36^. have reported that the same genotype has wide resistance to *M. graminicola*. Likewise, CS/ Synthetic (6X) and Tadinia showed specific resistance to two (33.3%) and one *Z. tritici* isolates, respectively, indicating that they could also be used as sources of resistance, and thus increase the genetic basis of wheat resistance to STB in Ethiopia. The majority of the wheat differential lines studied with known *Stb* genes were highly susceptible to Ethiopian *Z. tritici* populations. *Stb*2–*Stb*7 were ineffective against the Ethiopian *Z. tritici* Pathotype, and the differential lines Israel 493, Veranopolis, Tadinia, Shafir and Estanzuela Federal offered no protection against one or a few *Z. tritici* isolates. These findings are in line with previous reports by^28,37^, who identified these differential lines as susceptible to most *Z. tritici* isolates in Iran. TE9111, which carries the major gene *Stb11*, exhibited a 50% isolate-specific resistance, and thus represents a key resistance gene for Ethiopian *Z. tritici* isolates. This is in accordance with a study by^20^, who demonstrated that Quantitative trait loci (QTL) associated with STB resistance as identified by Genome wide association studies (GWAS) is mapped on chromosome 1B. Another study has mapped *Stb11* on the short arm of chromosome 1B in the TE9111 genotype^38^.

The analyses revealed high levels of diversity in the aggressiveness of the *Z. tritici* isolates tested (Fig. 1). ZSE158 was the most aggressive, with a mean PC of 61.4% and NLA of 54%. These results are comparable with the findings of^31^, who reported the highest mean PC and NLA (58%), for an isolate collected from the Bale Administrative zone in Ethiopia. The small differences observed between these two studies could be because of isolation and/or differential line variations. The Oromia region’s Arsi-Bale area is known as a ‘wheat belt’ because it produces the most wheat in Ethiopia. On susceptible wheat varieties planted in hot-spot areas in Ethiopia, STB has caused yield losses of up to 82%^39^. Additionally, recent research has revealed that STB causes yield losses of up to 41% and 48%, respectively, at agricultural research centers in Holeta^40^ and Areka^41^ in Ethiopia. Conversely, isolate ZSET218 appears to be the least aggressive, indicating that it may have an avirulence gene that is recognized by a common resistance gene found in the majority of the tested wheat genotypes. A study conducted by^30^ in Ethiopia reported that most *Z. tritici* isolates were avirulent, which contrasts with the findings of the current study, where the majority of Ethiopian isolates were virulent on both the tested genotypes and differential lines. This observation is, however, consistent with the findings of a previous report by^31^. The discrepancy may be explained by the genetic composition of the isolates either the earlier isolates produced multiple avirulence effectors recognized by resistance genes in the differential lines, or they carried a specific avirulence gene that was targeted by a resistance gene commonly present across several of the differential lines^34^.

Pathogen evolution is a major challenge in the management of plant diseases, especially in systems relying heavily on genetic resistance. *Zymoseptoria tritici*, the causal agent of Septoria tritici blotch (STB), is known for its high evolutionary potential. This is largely due to its mixed reproductive system (both sexual and asexual reproduction), high gene flow, large population sizes, and ability to rapidly adapt under selection pressure. When resistant wheat varieties carrying Stb genes are deployed over large areas, they exert strong selective pressure on the pathogen population. As a result, virulent strains capable of overcoming these resistance genes can emerge and become dominant, a phenomenon known as resistance breakdown. Over time, the effectiveness of resistance genes like Stb6, Stb11, or others may diminish as matching virulent Pathotype evolve. Studies such by^42,43^ have documented such breakdowns and emphasized the role of genetic diversity and recombination in *Z. tritici*. This underlines the importance of using resistance genes in combination (pyramiding), integrating them with quantitative resistance, and rotating cultivars to reduce the selection pressure on any single resistance gene.

To mitigate resistance breakdown, breeding programs must stay ahead of pathogen evolution by monitoring virulent shifts in pathogen populations, avoiding monocultures of single-resistance cultivars and promoting gene deployment strategies across different agro-ecological zones. Ultimately, a deeper understanding of pathogen evolution will inform more resilient breeding strategies and sustainable disease control measures.

## Conclusions

Wheat cultivation in Ethiopia is constrained considerably by several fungal diseases, including STB, which is caused by the hemibiotrophic fungus *Z. tritici.* Resistance breeding is the most efficient, cost-effective and environmentally friendly approach to managing STB, but developing sustainable management through a breeding program requires a clear understanding of the pathogen’s infection behavior and thorough screening of the available germplasm for potential resistance sources^44^. This research offers valuable data for Ethiopian breeding programs with the aim of initiating resistance against the devastating wheat disease STB.

This study’s findings offer important information regarding the resistance patterns of wheat differential lines against *Z. tritici* isolates recovered from the major wheat-growing areas of Ethiopia. This study has also profiled the virulence pattern of isolates. Ethiopian *Z. tritici* isolates exhibit broad pathogenicity against previously reported *Stb* genes, including *Stb2*–*Stb7*, suggesting that the *Z. tritici* populations may have successfully evolved to overcome these resistance genes through pathogenic host adaptation. Among the tested wheat differential lines, TE9111, which carries *Stb11*, showed resistance to 50% of the tested *Z. tritici* isolates, suggesting that it could be a good candidate source for wheat resistance breeding against STB. Overall, the broad virulence pattern of Ethiopian *Z. tritici* isolates indicates the need to look for possible sources of resistance and deploy them in susceptible but high yielding wheat genotypes. The insights provided into the infection behavior of *Z. tritici* isolates recovered from major wheat-growing areas of Ethiopia can inform development programs for STB-resistant wheat cultivars and efficient management plans within agricultural environments. Greater efforts should be directed toward identifying and characterizing resistance gene resources through both conventional and molecular approaches to effectively manage Septoria tritici blotch (STB) in Ethiopia. Additionally, more attention should be given to understanding the evolutionary potential of the pathogen, particularly its capacity to overcome genetic resistance over time.

Generally, this study highlights the complexity of host-pathogen interactions in *Z. tritici* and underscores the importance of strategic breeding and disease management to combat Septoria tritici blotch (STB) in Ethiopia. The observed variability in *Stb* gene efficacy and the high Pathotype diversity of the pathogen emphasize the need for a diversified approach to resistance breeding. Future research should focus on identifying novel resistance sources, monitoring pathogen evolution, and optimizing breeding strategies to ensure sustainable wheat production in the region.

## Materials and methods

### Determining the pathogenicity and virulence spectrum of *Z. tritici* isolates

Virulence analyses of the pathogen *Zymoseptoria tritici* were performed at the Swedish University of Agricultural Sciences (SLU, Alnarp, Sweden). The pathogen was shipped for use in a stock culture maintained in 25% glycerol. The cultures were plated in Petri dishes on potato dextrose agar (PDA) and incubated at 24 °C for 8 days. Single-spore-derived colonies were multiplied in liquid medium for 2 weeks for use as inoculum. Two hundred *Z. tritici* isolates which were used in previous study^45^ were molecularly identified by sequencing an internal transcribed spacer (ITS) rDNA region of 760 bp (Table 5), and a phylogenetic tree was generated. Based on the generated tree, six isolates (Table 6; Fig. 2) were selected for the virulence variability study. The infection behavior of the fungal isolates was tested on eight wheat differential lines with known *Stb* genes (obtained from SLU; Table 7) through artificial inoculation at the seedling stage under greenhouse conditions [with temperatures of 22 °C/21°C (day/night), a 12-hour photoperiod and relative humidity of 25–80%] at Biotron (Department of Plant Breeding, SLU, Alnarp, Sweden). Five seeds of each of the differential lines were planted in plastic pots (12 cm in diameter and 15 cm in depth) arranged in a factorial randomized complete block design (RCBD) and with four replicates. The soil type used in the study was potting soil clay and silica with a nutritional composition (g/m^3^) of nitrogen 182, phosphorus, 91, potassium, 194, magnesium, 274, sulfur, 99, calcium, 2186, iron, 8.6, manganese, 3.2), copper, 2.0, zinc, 1.0, boron, 0.4 and molybdenum, 2.6, and a pH of 5.5–6.5 (produced by SW Horto Co, Herrestadsvagen 24, Sweden).

**Table 5:** The ITS region primer pairs used for DNA fingerprinting of the *Zymoseptoria tritici* isolates.

Locus	Primer	Orientation	Sequence (5′ to 3′)	Length	Reference
ITS	ITS5	Forward	TCCTCCGCTTA TTGATATGC	760 bp	^46^
ITS	ITS4	Reverse	GGAAGTAAAAGTCGTAACAAGG		

**Table 6:** The six *Zymoseptoria tritici* isolates utilized in physiological race analyses and *Stb* gene efficacy testing.

Z. tritici isolates	Sequence (5′ to 3′)	Collection area		Geographic position (Universal Transverse Mercator coordinate system, UTM)	
**Name**	Forward	**Zone**	**District**	**Longitude**	Latitude
ZSET206	TTGCTCACGGGGGCGACCCTGCCGGGCGCCCCCGGAGGACCACCAAAAAACACTGCATCTCTGCGTCGGAGTTTACGAGTAAATCGAAACAAAACTTTCAACAACGGATCTCTTGGTTCTGGCATCGATGAAGAACGCAGCGAAATGCGATAAGTAATGTGAATTGCAGAATTCAGTGAATCATCGAATCTTTGAACGCACATTGCGCCCCCTGGTATTCCGGGGGGCATGCCCGTTCGAGCGTCATTACACCACTCCAGCCTCGCTGGGTATTGGGCGTCTTTTCGCGGGGGATCACTCCCCCGCGCGCCTCAAAGTCTCCGGCTGAGCGGTCTCGTCTCCCAGCGTTGTGGCATCACGTCTCGCCGCGGAGTTCACGAGCCCTCACGGCCGTTAAATCACACCTCAGGTTGACCTCGGATCGGGTAGGGATACCCGCTGAACTTAAACAT	West Shewa	Guder	09°02′630	037°44′785
ZSET218	GGGACATTACCGAGCGAGGGCCTCCGGGTCCGACCTCCAACCCTTTGTGAACACATCCCGTTGCTTCGGGGGCGACCCTGCCGGGCGCCCCCGGAGGACCACCAAAAAACACTGCATCTCTGCGTCGGAGTTTACGAGTAAATCGAAACAAAACTTTCAACAACGGATCTCTTGGTTCTGGCATCGATGAAGAACGCAGCGAAATGCGATAAGTAATGTGAATTGCAGAATTCAGTGAATCATCGAATCTTTGAACGCACATTGCGCCCCCTGGTATTCCGGGGGGCATGCCCGTTCGAGCGTCATTACACCACTCCAGCCTCGCTGGGTATTGGGCGTCTTTTCGCGGGGGATCACTCCCCCGCGCGCCTCAAAGTCTCCGGCTGAGCGGTCTCGTCTCCCAGCGTTGTGGCATCACGTCTCGCCGCGGAGTTCACGAGCCCTCACGGCCGTTAAATCACACCTCAGGTTGACCTCGGATCGGGTAGGGATACCCGCTGAACTTAAGCATATCAAAA	North Shewa	Kuyu	0958′ 761	038°86′ 109
ZSET121	GGAGATCATTATTGATTGGTCGAAAGACCTTATCAGATTCTACCACCTCTGTGAACCGTTGACCTCCGGGTTAATAATCAAACATCAGTGTAACGAACGTAAGAGTATCTTAATTAAACAAAACTTTTAACAACGGATCTCTTGGCTCTCGCATCGATGAAGAACGCAGCGAAATGCGATAAGTAATGTGAATTGCAGAATTCAGTGAATCATCGAATCTTTGAACGCACCTTGCGCCTTTTGGTATTCCGAAAGGCATGCCTGTTTCAGTGTCATGAAATCTCAATCTAATATGTTTTCTGAACATGTTAGGCTTGGACTTGGGTGTCTGCCAGCAATGGCTCACCTCAAATGACTTAGTGGAACATCCCACATCAGTGTTAGACGTAATAAGTTTCGTCTCTCCTTGTGGTGATGACTGCTCAAAACCTGCCATCGCGCACCTTTTGACTTTGACCTGAAATCAGGTAGGGCTACCCGCTGAACTTAAGCATATCAAAA	Arsi	Mararo	07°40′726	039°24′889
ZSET033	AACCTCCCAACCCTTTTGTGAACACATCCCGTTGCTTCGGGGGCGACCCTGCCGGGCGCCCCCGGAGGACCACCAAAAAACACTGCATCTCTGCGTCGGAGTTTACGAGTAAATCGAAACAAAACTTTCAACAACGGATCTCTTGGTTCTGGCATCGATGAAGAACGCAGCGAAATGCGATAAGTAATGTGAATTGCAGAATTCAGTGAATCATCGAATCTTTGAACGCACATTGCGCCCCCTGGTATTCCGGGGGGCATGCCCGTTCGAGCGTCATTACACCACTCCAGCCTCGCTGGGTATTGGGCGTCTTTTCGCGGGGGATCACTCCCCCGCGCGCCTCAAAGTCTCCGGCTGAGCGGTCTCGTCTCCCAGCGTTGTGGCATCACGTCTCGCCGCGGAGTTCACGAGCCCTCACGGCCGTTAAATCA	OSZ	Walmara	09°05′654	038°50′724
ZSET168	TTGCTTCGGGGGCGACCCTGCCGGGCGCCCCCGGAGGACCACCAAAAAACACTGCATCTCTGCGTCGGAGTTTACGAGTAAATCGAAACAAAACTTTCAACAACGGATCTCTTGGTTCTGGCATCGATGAAGAACGCAGCGAAATGCGATAAGTAATGTGAATTGCAGAATTCAGTGAATCATCGAATCTTTGAACGCACATTGCGCCCCCTGGTATTCCGGGGGGCATGCCCGTTCGAGCGTCATTACACCACTCCAGCCTCGCTGGGTATTGGGCGTCTTTTCGCGGGGGATCACTCCCCCGCGCGCCTCAAAGTCTCCGGCTGAGCGGTCTCGTCTCCCAGCGTTGTGGCATCACGTCTCGCCGCGGAGTTCACGAGCCCTCACGGCCGTTAAATCACACCTCAGGTTGACCTCGGATCGGGTAGGGATACCCGCTGAACTTAAGCATATCAATAAGCGGAGGAACTGCAC	South-west Shewa	Waliso	08°63′242	038°04′1451
ZSET158	TTTGCTTCGGGGGCGACCCTGCCGGGCGCCCCCGGAGGACCACCAAAAAACACTGCATCTCTGCGTCGGAGTTTACGAGTAAATCGAAACAAAACTTTCAACAACGGATCTCTTGGTTCTGGCATCGATGAAGAACGCAGCGAAATGCGATAAGTAATGTGAATTGCAGAATTCAGTGAATCATCGAATCTTTGAACGCACATTGCGCCCCCTGGTATTCCGGGGGGCATGCCCGTTCGAGCGTCATTACACCACTCCAGCCTCGCTGGGTATTGGGCGTCTTTTCGCGGGGGATCACTCCCCCGCGCGCCTCAAAGTCTCCGGCTGAGCGGTCTCGTCTCCCAGCGTTGTGGCATCACGTCTCGCCGCGGAGTTCACGAGCCCTCACGGCCGTTAAATCACACCTCAGGTTGACCTCGGATCGGGTAGGGATACCCGCTGAACTTAAGCATATCAAAA	West Arsi	Kofale	08°12′958	039°36′148

*OSZ* Oromia special Zone Finfinne area, *UTM* Universal Transverse Mercator coordinate system.

**Table 7:** The eight wheat differential lines with known *Stb* genes used for virulence variability testing.

No	Genotype	Chromosome	Origin	Growing habit	Stb gene(s)	Reference
1	Veranopolis	1BS	Brazil	Spring	*Stb2* + *Stb6*	^47^
2	Israel 493	7AS	Israel	Spring	*Stb3* + *Stb6*	^47^
3	Tadinia	7DS	USA	Spring	*Stb4* + *Stb6*	^48^
4	CS/(synthetic (6x)	7DS	China/USA	Spring	*Stb5* + *Stb6*	^49^
5	Shafir (*stb*6)	3AS	Israel	Spring	*Stb6*	^50^
6	Estanzuela Federal (*stb*7)	4AL	Uruguay	Spring	*Stb7*	^51^
7	TE9111	1BS	Portugal	Spring	*Stb6*,*Stb7 and Stb11*	^52^
8	Taichung 29		Japan	Spring	Susceptible control	

#### Seedling inoculation

Single spore-derived colonies were transferred into a liquid medium composed of 1% (w/v) yeast extract powder + 1% (w/v) sucrose, and cultures were maintained in an orbital shaker at 130 rpm for 2 weeks for spore multiplication. Spore pellets were then recovered by centrifugation at 10,000 rpm for 5 min. The pellets were suspended in distilled sterilized water, and the spore concentration adjusted to 10^7^ spore/ml using hemacytometer. The solution was supplemented with 0.15% Tween 20 (polyoxyethylene – sorbitan monolaurate; Sigma-Aldrich, Poznan, Poland), and 10 µl (10^7^ spore/ml) of mono-spore suspensions of the individual isolates were hand sprayed until run-off^32^. To avoid cross-contamination, inoculated plantlets were covered with polyethylene plastic bags.

### Data collection and disease evaluation

Wheat differential lines carrying distinct Septoria tritici blotch (STB) resistance genes (*Stb2*–*Stb11*, excluding *Stb*8 and *Stb*9) were used to analyze the virulence variability of the Ethiopian *Z. tritici* isolates. The susceptible wheat variety Taichung 29 was used as a baseline for the virulence spectra of the isolates and the efficacy of the genes. The responses of the wheat genotypes were evaluated at the seedling stage under greenhouse conditions as described by^31^, with minor modifications, and the plants were monitored for symptom development for a period of 3 weeks. Based on extensive research performed on interactions between *Z. tritici* isolates and host cultivars^29^, two parameters were used to assess disease severity: the percentage of necrotic leaf area (NLA) and the percentage of pycnidia coverage (PC). Disease severity scoring was carried out at 21 days post-inoculation (dpi) on the second leaf of 15 plants per isolate–genotype combination, by visual estimation of the %NLA and %PC. The values were averaged per pot for further analysis. The percentage data was then converted into a scale of 0–5^53^, where 0 (Immune - Imm) (0%): No pycnidial formation, with no symptoms or only occasional hypersensitive flecks, 1 (Highly Resistant - HR) (5–10%): No or very few isolated pycnidia, mainly in older leaf tissue, with hypersensitive flecking in younger leaves, 2 (Resistant - R) (11–20%): Very light pycnidial formation, 3 (Intermediate - I) (21–29%): Light pycnidial formation with noticeable lesion coalescence, especially towards the leaf tip and in older leaf tissue, 4 (Susceptible - S) (30–50%): Moderate pycnidial formation with significant lesion coalescence and 5 (Very Susceptible - VS) (51% and above): Large, abundant pycnidia with extensive lesion coalescence ^16,54^.

### Data analysis

In studies on the interaction between various Z. tritici isolates and host cultivars, disease severity was estimated using the percentage of leaf area with necrosis, pycnidia coverage, and their combinations^16^. The percentage data were transformed using the arcsine method and the generalized linear model was used to examine the normalized data in order to determine the source of variance (ANOVA) using SAS software version 9.4 (SAS Institute, Cary, NC, USA). The effects of isolate, wheat cultivar, and their two-way interactions, were considered to be fixed effects, and the block effect as random effect. Significant means were separated using the Tukey procedure at the α = 5% significance level^55^. Specific interactions between wheat genotypes and pathogen isolates were determined by computing the least significant differences (LSD) of means of wheat genotype-by-isolate interactions at α = 1% and 5% significance levels^33,34^. The interaction means values lower than the LSD values at α = 1% and 5% significant levels were considered as resistant and highly resistant genotypes, respectively. Mean disease severity values of the genotypes (differential lines)-by-isolate were subjected to a hierarchical cluster analysis. The analyses were performed using a hierarchical clustering method^56^, and a dissimilarity matrix was measured using Ward’s method implemented in JMP pro17 (SAS Institute).

## Supplementary Information

Below is the link to the electronic supplementary material.

Supplementary Material 1

Supplementary Material 2

Supplementary Material 3

## Data Availability

The data that support the study are in the article and supplementary materials. Sequence data has been deposited at the National Centre for Biotechnology Information (NCBI) under the accession SUB14926029: PQ755050 - PQ755210.
