# Supplementary material for: Efficacy of Stb resistance genes and pathotype diversity in Zymoseptoria tritici from Ethiopia
Source: Sci Rep. 2025 Jul 31;15:28030. doi: 10.1038/s41598-025-13035-x (PMC12314099; doi:10.1038/s41598-025-13035-x)
Supplement: Supplementary file 1 — Supplementary Material 1 [file 41598_2025_13035_MOESM1_ESM.pdf]

Supplementary Table 1. Mean Interaction Data for PC and NLA of Eight (8) Genotype and Six (6) Z.tritici Isolates used for Clustering Analysis

Number	Genotype-Isolate interaction	PC LSMEAN	NLA LSMEAN
1	Estanzuela-ZSET033	47.5	37
2	Estanzuela-ZSET121	58.25	45.75
3	Estanzuela-ZSET158	60	49
4	Estanzuela-ZSET168	48	38
5	Estanzuela-ZSET206	50.25	39.25
6	Estanzuela-ZSET218	42	32.75
7	Israel-ZSET033	63.75	59
8	Israel-ZSET121	40.75	38.25
9	Israel-ZSET158	72.5	64
10	Israel-ZSET168	60.5	54.75
11	Israel-ZSET206	51.25	43.75
12	Israel-ZSET218	63	60.75
13	Shafir-ZSET033	58.5	49.75
14	Shafir-ZSET121	66.25	59.25
15	Shafir-ZSET158	71.5	60.5
16	Shafir-ZSET168	64.5	49
17	Shafir-ZSET206	65	57.75
18	Shafir-ZSET218	62.5	54
19	TE9111-ZSET033	26.25	18.75
20	TE9111-ZSET121	31.75	25.75
21	TE9111-ZSET158	17.5	11
22	TE9111-ZSET168	10.75	6.75
23	TE9111-ZSET206	30	19.75
24	TE9111-ZSET218	13.25	8
25	Tadinia-ZSET033	55.25	50.25
26	Tadinia-ZSET121	58	51
27	Tadinia-ZSET158	66.5	61.25
28	Tadinia-ZSET168	56.25	48.25
29	Tadinia-ZSET206	63.25	60.25
30	Tadinia-ZSET218	39	23.75

31	Taichung-ZSET033	85	80.25
32	Taichung-ZSET121	84.5	84
33	Taichung-ZSET158	85	77.5
34	Taichung-ZSET168	76.25	71.25
35	Taichung-ZSET206	82.5	83
36	Taichung-ZSET218	81.25	78.5
37	Veranopos-ZSET033	60.5	50.75
38	Veranopos-ZSET121	53.75	49.5
39	Veranopo-ZSET158	67	57.75
40	Veranopos-ZSET168	57.5	46.25
41	Veranopo-ZSET218	60.75	60.75
42	Veranopos-ZSET206	42.5	40.25
43	synthetic-ZSET033	31.25	23.25
44	synthetic-ZSET121	45.25	37
45	synthetic-ZSET158	64.25	52
46	synthetic-ZSET168	67.25	57
47	synthetic-ZSET206	45	37
48	synthetic-ZSET218	27	20.5

PC = Pycnidia Coverage, NLA = Necrotic Leaf Area

Supplementary Table 2. Raw data used for the analysis of Pathotype Efficacy of Ethiopian *Z.tritici* Isolates

Observation	Genotype	Replication	Isolate	%NLA	% PC
1	synthetic	1	ZSET158	50	65
2	synthetic	1	ZSET168	55	60
3	synthetic	1	ZSET033	20	30
4	synthetic	1	ZSET121	40	45
5	synthetic	1	ZSET206	45	50
6	synthetic	1	ZSET218	15	20
7	synthetic	2	ZSET158	50	60
8	synthetic	2	ZSET168	65	67

9	synthetic	2	ZSET033	15	25
10	synthetic	2	ZSET121	48	50
11	synthetic	2	ZSET206	40	45
12	synthetic	2	ZSET218	25	30
13	synthetic	3	ZSET158	53	65
14	synthetic	3	ZSET168	63	70
15	synthetic	3	ZSET033	28	35
16	synthetic	3	ZSET121	35	40
17	synthetic	3	ZSET206	30	50
18	synthetic	3	ZSET218	22	28
19	synthetic	4	ZSET158	55	67
20	synthetic	4	ZSET168	65	72
21	synthetic	4	ZSET033	30	35
22	synthetic	4	ZSET121	25	46
23	synthetic	4	ZSET206	33	35
24	synthetic	4	ZSET218	25	30
25	Estanzuela	1	ZSET158	38	45
26	Estanzuela	1	ZSET168	40	50
27	Estanzuela	1	ZSET033	38	48
28	Estanzuela	1	ZSET121	48	55
29	Estanzuela	1	ZSET206	38	50
30	Estanzuela	1	ZSET218	23	35
31	Estanzuela	2	ZSET158	40	50
32	Estanzuela	2	ZSET168	40	45
33	Estanzuela	2	ZSET033	35	45
34	Estanzuela	2	ZSET121	55	60
35	Estanzuela	2	ZSET206	33	45
36	Estanzuela	2	ZSET218	35	40
37	Estanzuela	3	ZSET158	40	45

38	Estanzuela	3	ZSET168	42	55
39	Estanzuela	3	ZSET033	33	45
40	Estanzuela	3	ZSET121	45	58
41	Estanzuela	3	ZSET206	38	51
42	Estanzuela	3	ZSET218	30	40
43	Estanzuela	4	ZSET158	48	50
44	Estanzuela	4	ZSET168	30	42
45	Estanzuela	4	ZSET033	42	52
46	Estanzuela	4	ZSET121	35	60
47	Estanzuela	4	ZSET206	48	55
48	Estanzuela	4	ZSET218	43	53
49	Israel	1	ZSET158	63	70
50	Israel	1	ZSET168	58	65
51	Israel	1	ZSET033	60	65
52	Israel	1	ZSET121	65	68
53	Israel	1	ZSET206	35	40
54	Israel	1	ZSET218	63	65
55	Israel	2	ZSET158	65	75
56	Israel	2	ZSET168	48	52
57	Israel	2	ZSET033	35	45
58	Israel	2	ZSET121	45	50
59	Israel	2	ZSET206	40	55
60	Israel	2	ZSET218	60	62
61	Israel	3	ZSET158	60	72
62	Israel	3	ZSET168	50	55
63	Israel	3	ZSET033	68	70
64	Israel	3	ZSET121	0	0
65	Israel	3	ZSET206	50	55
66	Israel	3	ZSET218	65	68

67	Israel	4	ZSET158	68	73
68	Israel	4	ZSET168	63	70
69	Israel	4	ZSET033	73	75
70	Israel	4	ZSET121	43	45
71	Israel	4	ZSET206	50	55
72	Israel	4	ZSET218	55	57
73	Shafir(s	1	ZSET158	68	75
74	Shafir(s	1	ZSET168	33	55
75	Shafir(s	1	ZSET033	50	60
76	Shafir(s	1	ZSET121	68	75
77	Shafir(s	1	ZSET206	63	65
78	Shafir(s	1	ZSET218	53	60
79	Shafir(s	2	ZSET158	63	73
80	Shafir(s	2	ZSET168	53	58
81	Shafir(s	2	ZSET033	53	58
82	Shafir(s	2	ZSET121	58	65
83	Shafir(s	2	ZSET206	65	70
84	Shafir(s	2	ZSET218	50	65
85	Shafir(s	3	ZSET158	53	68
86	Shafir(s	3	ZSET168	60	80
87	Shafir(s	3	ZSET033	48	56
88	Shafir(s	3	ZSET121	53	62
89	Shafir(s	3	ZSET206	65	70
90	Shafir(s	3	ZSET218	60	65
91	Shafir(s	4	ZSET158	58	70
92	Shafir(s	4	ZSET168	50	65
93	Shafir(s	4	ZSET033	48	60
94	Shafir(s	4	ZSET121	58	63
95	Shafir(s	4	ZSET206	38	55

96	Shafir(s	4	ZSET218	53	60
97	Tadinia	1	ZSET158	55	60
98	Tadinia	1	ZSET168	43	50
99	Tadinia	1	ZSET033	48	52
100	Tadinia	1	ZSET121	68	70
101	Tadinia	1	ZSET206	55	60
102	Tadinia	1	ZSET218	10	15
103	Tadinia	2	ZSET158	60	63
104	Tadinia	2	ZSET168	50	55
105	Tadinia	2	ZSET033	58	60
106	Tadinia	2	ZSET121	40	52
107	Tadinia	2	ZSET206	58	60
108	Tadinia	2	ZSET218	20	45
109	Tadinia	3	ZSET158	60	68
110	Tadinia	3	ZSET168	50	65
111	Tadinia	3	ZSET033	40	45
112	Tadinia	3	ZSET121	38	40
113	Tadinia	3	ZSET206	63	65
114	Tadinia	3	ZSET218	30	48
115	Tadinia	4	ZSET158	70	75
116	Tadinia	4	ZSET168	50	55
117	Tadinia	4	ZSET033	55	64
118	Tadinia	4	ZSET121	58	70
119	Tadinia	4	ZSET206	65	68
120	Tadinia	4	ZSET218	35	48
121	Taichung	1	ZSET158	83	90
122	Taichung	1	ZSET168	70	75
123	Taichung	1	ZSET033	83	85
124	Taichung	1	ZSET121	88	90

125	Taichung	1	ZSET206	72	75
126	Taichung	1	ZSET218	83	85
127	Taichung	2	ZSET158	80	85
128	Taichung	2	ZSET168	65	70
129	Taichung	2	ZSET033	70	80
130	Taichung	2	ZSET121	80	75
131	Taichung	2	ZSET206	95	90
132	Taichung	2	ZSET218	83	85
133	Taichung	3	ZSET158	72	80
134	Taichung	3	ZSET168	70	75
135	Taichung	3	ZSET033	88	90
136	Taichung	3	ZSET121	83	85
137	Taichung	3	ZSET206	80	85
138	Taichung	3	ZSET218	78	80
139	Taichung	4	ZSET158	75	85
140	Taichung	4	ZSET168	80	85
141	Taichung	4	ZSET033	80	85
142	Taichung	4	ZSET121	85	88
143	Taichung	4	ZSET206	85	80
144	Taichung	4	ZSET218	70	75
145	TE9111	1	ZSET158	8	15
146	TE9111	1	ZSET168	10	15
147	TE9111	1	ZSET033	10	20
148	TE9111	1	ZSET121	25	30
149	TE9111	1	ZSET206	18	25
150	TE9111	1	ZSET218	5	8
151	TE9111	2	ZSET158	8	10
152	TE9111	2	ZSET168	5	8
153	TE9111	2	ZSET033	15	25

154	TE9111	2	ZSET121	30	35
155	TE9111	2	ZSET206	18	25
156	TE9111	2	ZSET218	5	10
157	TE9111	3	ZSET158	13	20
158	TE9111	3	ZSET168	10	15
159	TE9111	3	ZSET033	25	30
160	TE9111	3	ZSET121	28	30
161	TE9111	3	ZSET206	18	30
162	TE9111	3	ZSET218	10	15
163	TE9111	4	ZSET158	15	25
164	TE9111	4	ZSET168	2	5
165	TE9111	4	ZSET033	25	30
166	TE9111	4	ZSET121	20	32
167	TE9111	4	ZSET206	25	40
168	TE9111	4	ZSET218	12	20
169	Veranopolis	1	ZSET158	68	75
170	Veranopolis	1	ZSET168	40	52
171	Veranopolis	1	ZSET033	43	65
172	Veranopolis	1	ZSET121	45	45
173	Veranopolis	1	ZSET206	73	55
174	Veranopolis	1	ZSET218	20	15
175	Veranopolis	2	ZSET158	55	65
176	Veranopolis	2	ZSET168	50	65
177	Veranopolis	2	ZSET033	45	52
178	Veranopolis	2	ZSET121	50	55
179	Veranopolis	2	ZSET206	60	68
180	Veranopolis	2	ZSET218	40	47
181	Veranopolis	3	ZSET158	40	58
182	Veranopolis	3	ZSET168	55	60

183	Veranopolis	3	ZSET033	50	55
184	Veranopolis	3	ZSET121	60	65
185	Veranopolis	3	ZSET206	55	60
186	Veranopolis	3	ZSET218	53	58
187	Veranopolis	4	ZSET158	68	70
188	Veranopolis	4	ZSET168	40	53
189	Veranopolis	4	ZSET033	65	70
190	Veranopolis	4	ZSET121	43	50
191	Veranopolis	4	ZSET206	55	60
192	Veranopolis	4	ZSET218	48	50

%PC = Percentage Pycnidia Coverage, %NLA = Percentage Necrotic Leaf Area
