# Supplementary material for: Efficacy of Stb resistance genes and pathotype diversity in Zymoseptoria tritici from Ethiopia
Source: Sci Rep. 2025 Jul 31;15:28030. doi: 10.1038/s41598-025-13035-x (PMC12314099; doi:10.1038/s41598-025-13035-x)
Supplement: Supplementary file 3 — Supplementary Material 3 [file 41598_2025_13035_MOESM3_ESM.pdf]

Supplementary Table 1. Mean Interaction Data for PC and NLA of Eight (8) Genotype and Six (6) Z.tritici Isolates used for Clustering Analysis

Supplementary Table 2. Raw data used for the analysis of Pathotype Efficacy of Ethiopian *Z.tritici* Isolates
